# Charge Nurse Perspectives on Frontline Leadership in Acute Care Environments

**DOI:** 10.5402/2011/164052

**Published:** 2011-11-16

**Authors:** Rose O. Sherman, Ruth Schwarzkopf, Anna J. Kiger

**Affiliations:** ^1^Christine E. Lynn College of Nursing, Florida Atlantic University, 777 Glades Road CON 121, Boca Raton, FL 33431, USA; ^2^West Boca Medical Center, 21644 State Road 7, Boca Raton, FL 33428, USA; ^3^Tenet Healthcare Corporation, 1445 Ross Avenue, Dallas, TX 75202, USA

## Abstract

A recently issued report from the Institute of Medicine (IOM) in the United States on the *Future of Nursing* included a recommendation that nurses should receive leadership development at every level in order to transform the healthcare system. Charge nurses, at the frontline of patient care in acute care settings, are in key positions to lead this change. This paper presents findings from research conducted with nurses in the Tenet Health System. Charge nurses from ten facilities who attended a one-day work shop were surveyed to gain insight into the experience of being a frontline leader in today's acute care environment. The relationship of these findings to the IOM report and the implications for both the Tenet Health System and other healthcare organizations that are working to support nurses who assume these challenging roles are discussed.

## 1. Introduction

The recent Institute of Medicine (IOM) report on *The Future of Nursing: Leading Change, Advancing Health *[1] includes a recommendation that nurses should be prepared and enabled to lead change to advance healthcare in the United States. It is noted in the report that strong leadership at every level is critical if the vision of a transformed health care system is to be realized, and nurses are to participate as full partners with physicians and other health professionals. In the current environment, nurses are often placed in leadership situations without the needed competencies and skills to meet these challenges and other important organizational imperatives. This is especially true for professional nurses who are asked to assume frontline leadership roles such as that of charge nurse. With rising patient acuity, decreased lengths of stay, staffing shortages, pay for performance measures and new technologies, the context of health care environments has significantly changed, and these roles have become more complex. Porter-O'Grady and Malloch [2] have noted that the demands of leadership change as the world changes. Stepping back and reconsidering the skill sets and competencies that leaders need is an important exercise in planning for the future. 

The nurse executive leadership of Tenet Healthcare, who has responsibility for 49 acute care facilities and one long-term care facility in the United States, has committed to a journey to develop frontline nurse leaders. Charge nurses from ten facilities who attended a one-day work shop were surveyed to gain insight into the experience of being a frontline leader in today's acute care environment. The purpose of this paper is to present the findings of the study and relate them to recommendations in the IOM report [1]. The implications for both Tenet Healthcare and other healthcare organizations that are working to support nurses who assume these challenging roles are discussed. 

## 2. Review of the Literature

The focus of most nursing leadership literature and research has been on the roles, competencies, and impact of nurse managers and executives. Fewer articles have been written about charge nurses who assume frontline clinical leadership responsibilities at the unit level. In the 1970s, Hinkle and Hinkle [3] described the charge nurse role as overseeing patient care, providing complete documentation of care, and managing staff interactions. Over time, the charge nurse role became more complex and there were additional recommendations in the literature about the skill sets needed to be effective. Mahlmeister [4], an attorney and expert in professional accountability, recommended that charge nurses and assistant nurse managers should be well educated about their professional accountability and legal liability under nurse practice acts. Connelly et al. [5] in their qualitative study of charge nurse competencies described the importance of clinical, critical thinking, organizational, and human relations competencies. Kalisch et al. [6] found in their research that the team leadership skills of charge nurses is key to effective team functioning, and they often performed care that would otherwise be missed by team members. Jasper et al [7] identified three common themes in research done in Wales prior to the development of a program targeted at different levels of leadership including the charge nurse role. These included managing unit performance, managing people and resources, and empowerment of self and others. Admi and Moshe-Eilon [8] studied role stress among charge nurses in Israel. Managerial decision making, authority-responsibility conflict, overload, deficiency of resources, role conflict, and patient-nurse interaction were major stress factors.

Authors agree that charge nurses are expected to lead staff while managing the work systems and processes on their units to insure that the needs of patients are met. It is a skillful balancing act and not all organizations provide the type of leadership training that the charge nurse may need [9–12]. Positions such as that of the charge nurse or assistant nurse manager usually have limited formal leadership power. Their impact on patient care and outcomes is often less visible at the organizational level. Yet as their own administrative responsibilities have expanded, nurse managers increasingly depend on these frontline leaders to assume responsibility for quality outcomes and help meet the growing number of organizational performance measures. The aim of this research was to examine leadership qualities needed today in these roles, the challenges, and role satisfiers from the perspective of the frontline nurse leader. 

## 3. Methods

### 3.1. Study Design

**Figure 1:**
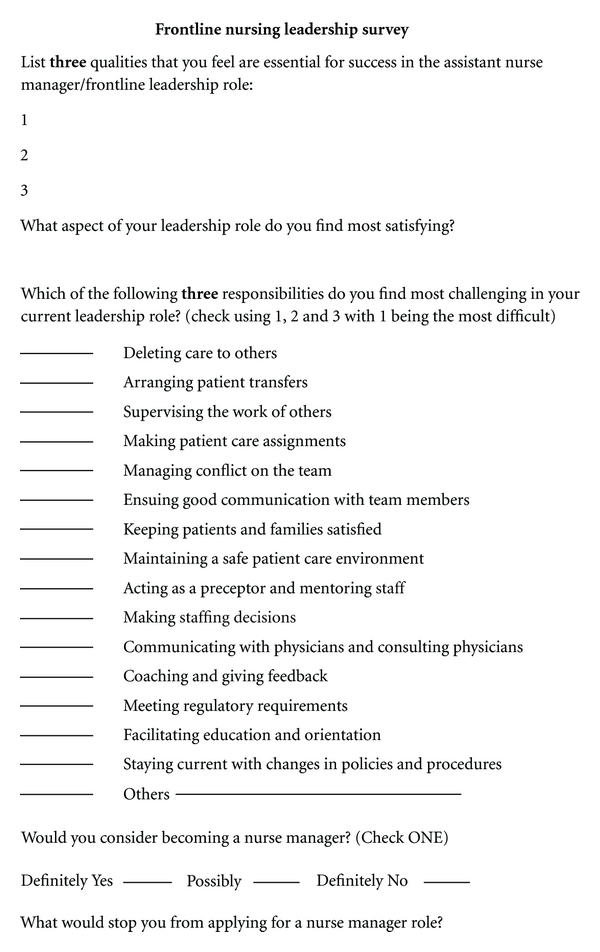
Frontline nursing leadership survey.

An exploratory descriptive design was used in this study. A survey with five open and closed-ended questions designed by the authors (Figure 1) was distributed to the 400 charge nurses who attended one of the ten workshops in the spring of 2010 sponsored by Tenet Healthcare. 

### 3.2. Sample

The convenience sample was used in this study. It included 400 charge nurses from 10 Tenet Healthcare facilities in South Florida who participated in a leadership development workshop. The criteria for selection for participation in the seminar were that the nurse needed to be in a full-time charge nurse role from any specialty area of the hospital with the potential for grooming to accept more responsibilities. Demographic information for the 354 study participants is presented in Table 1. The participants were experienced nurses with a mean age of 46.5 years and 19.5 years of work experience. More than half (55%) had either an associate degree or a diploma degree as their highest level of nursing education. They worked in a wide range of specialty areas in ten Tenet hospitals, and all participants worked in acute care hospital settings. 

### 3.3. Data Collection

Workshop participants were read information about the research and asked to complete a survey upon arrival to the educational seminar. Questions on the survey were used to begin discussion about the charge nurse role by the keynote speaker who was the first presenter on the program agenda. After this presentation, those who consented to participate in the study were asked to turn in their surveys and demographic information for data analysis. At the conclusion of the session, 354 charge nurses agreed to participate in the study for a survey return rate of 88%. Participants were asked about leadership qualities needed to be a frontline leader, their challenges, role satisfiers, and whether they would consider applying for a nurse manager position. Approval for study was given by the Investigational Review Board at Florida Atlantic University, and written permission was also received from the Tenet System Chief Nursing Officer.

### 3.4. Data Analysis

Frequencies were tabulated on the two close-ended questions. A content analysis was done on the written responses to the three open-ended questions. The data was initially analyzed for key words; then emerging themes were developed by the authors. The themes and study findings were reviewed with Tenet nurse leaders during a regional leadership meeting for validation. 

## 4. Findings

### 4.1. Qualities Needed by Frontline Leaders Today

When considering nurses for frontline leadership roles, those currently working in the role rated the ability to manage good communication as a critical quality for success (Table 2). During workshop discussions, participants noted that communication is challenging in today's environment with both the diversity in the workforce and the culturally diverse patient populations served by their hospitals. Good communication was viewed as being critical to patient safety. Charge nurses have to monitor the consistent use of communication tools that organizations have in place such as team huddles, bedside report, and hourly patient rounds. The role of the charge nurse was described as being similar to that of an air traffic controller. Charge nurses need to have strong organizational skills to organize the work of their teams. They also have to effectively manage their time and control their own stress levels. Clinical competence in the area assigned was identified as being important to effectively coach and mentor others. The quality of being approachable and nonjudgmental was seen as critical. Younger, less experienced staff need to feel safe when seeking help to avoid making errors.

### 4.2. Most Challenging Role Responsibilities

Participants identified many leadership challenges in their acute care environments (Table 3). Their most significant challenge is managing the conflict on their teams. This conflict can occur between nursing team members, with physicians or other departments. The generational diversity in the healthcare workforce presents new challenges for those in leadership roles. Charge nurses report significant differences among team members in values and beliefs about teamwork, loyalty, use of social networking, and preferred methods of communication. Some charge nurses commented that in the past, team discussions about work situations were usually confined to the workplace. Social networking sites such as Facebook have added a new dimension to relationships and discussions occurring among team members. The charge nurses observed that finding the time and having the skill to work through the many conflicts that occur in work environments is difficult. 

Patients and families today were noted to be informed consumers with higher expectations than in the past. Charge nurses spoke about the impact on their roles with the initiation of the *Hospital Care Quality Information from a Consumer Perspective *(HCAHPS) survey [13] that is now publicly reported in the United States and tied to reimbursement. They are aware of their responsibility and role in patient satisfaction as this data is reported at the unit level and there is direct accountability. Charge nurses are also involved with the monitoring of core measures recently initiated by the Centers for Medicare and Medicaid, nursing sensitive indicators such as pressure ulcer development, and the avoidance of never events such as patient falls on their shifts.

With frequent changes in regulatory requirements related to patient safety, pay for performance indicators, technology, and organizational expectations, charge nurses reported difficulty staying current with policies and procedures. Seasoned charge nurses observe that the pace of change is so rapid today that it is exhausting for frontline leaders. The delegation of care in high-acuity environments was also noted to be very challenging. Charge nurses need to carefully consider the competency level of their staff and the needs of the patient when making assignments. Knowing when to step in to help staff and when to step back to let them manage complex situations is a balancing act. 

### 4.3. Role Satisfiers

The frontline nurse leaders who attended these workshops were in permanent charge nurse roles. Nurse leaders report that it can be difficult to recruit nurses to assume these roles. Although there are challenges in the role, the frontline leaders report many satisfying aspects of their work. Seven themes emerged from a content analysis of their open-ended answer to the question about what they found most satisfying in the role (Table 4). The opportunity to develop staff was a theme that resonated with many charge nurses. They wrote about the joy they experienced when they watched new graduates develop into confident professionals. Although keeping patients and families happy was noted to be a challenge, the ability to keep patients happy and achieve good outcomes was also a strong satisfier. Their ability to effectively lead their teams and hearing positive feedback about their leadership from others provide role satisfaction. Charge nurses report that they enjoy knowing that they make a difference. Their skill in effectively solving problems, and reducing the fragmentation in their environments is critical to being effective in the role. In their responses, they talked about the fulfillment they felt in being able to manage the flow on their units, troubleshoot problems, and organize care. Some charge nurses reported that seeing their own growth in their leadership role led to a sense of achievement. Their ability to contribute to the maintenance of quality and keep their patients safe in today's chaotic healthcare environments gave them great satisfaction.

### 4.4. Factors Impacting Movement into Leadership Roles

Succession planning is a key concern in many healthcare organizations. When seeking to fill nurse manager roles, nurse leaders often consider their charge nurses as excellent potential candidates. As is true with many healthcare organizations, nurse leaders in Tenet have found that recruitment for the nurse manager role poses challenges. In this study, only 34% of charge nurses indicated that they would definitely consider a nurse manager role, 46% would possibly consider the role, and 21% indicated that they definitely would not. The factors that would stop participants from applying are presented in Table 5. In their roles, the charge nurses appear to gain a strong appreciation of what is expected of their nurse managers. Some charge nurses reported that they currently make more than their managers with overtime and shift differentials. Many also felt they had more job security in their current roles and worked fewer hours. There was also concern about losing their clinical skills and connections with patients. Interestingly some charge nurses indicated that they would consider the role but deemed themselves too old or lacked the qualifications, education, or confidence. 

## 5. Discussion

### 5.1. Study Limitations

These study findings were generated from research conducted with charge nurses in one health system in the United States. Their perspectives about the role may not be generalizable to charge nurses in other settings within the US or internationally. At the time this study was done, the charge nurses surveyed were attending the initial session of a one-year leadership development program. It is possible with leadership development that the charge nurses could feel differently about their challenges and desire to move into a more formal leadership role. 

### 5.2. Leadership and Educational Implications

Frontline nurse leaders, such as those interviewed in this study, are in key positions to help transform the healthcare delivery system. The research findings indicate that in order to meet the many challenges that they face in their environments, charge nurses will need ongoing competency development. They also need encouragement to seek a baccalaureate degree as more than half of the nurses responding in this study were not baccalaureate prepared. Strong interdisciplinary teamwork is needed to promote change, but managing team conflict particularly with physicians was cited as a primary role challenge. The charge nurses surveyed in this study understand their role in ensuring quality care but also expressed frustration and an inability to stay current with rapid changes in technology, performance indicators, safety goals, and regulatory requirements. 

As is true in many health systems, leadership development in the Tenet Health System had not historically included nurses working at the frontlines of patient care. The corporate Chief Nursing Officer, working with the Nurse Executive Council, requested that the ten Tenet South Florida Chief Nursing Officers develop and conduct a one-day pilot program for frontline nurses in their facilities. The research presented in this paper was collected during the pilot program. Using participant feedback from the pilot program, the initiative will then be expanded to include additional classes and leadership experiences relevant to charge nurse professional education needs. Ultimately, the goal is to develop a model leadership program that can be rolled out to the 49 acute care hospitals in the corporation. 

The initial program received excellent feedback from participants. Using both the survey research conducted during the workshop and program evaluations, it was clear that the charge nurses both needed and wanted more classes. Their feedback provided direction about priorities for the next steps in initiative planning. Conflict management among team members, with physicians and other departments, was identified as both a major role challenge and the top future learning need by participants. Looking at the requests for future classes helped nurse leaders to step back and realize just how much conflict the charge nurses deal with every day. The conflicts arise with physicians, families, demanding patients, and, unfortunately, each other. Although this topic was covered in the one-day workshop, charge nurse participants clearly wanted more content. 

Future Tenet educational programs will include scenario-based simulation using real case studies of challenging situations submitted by the charge nurses and their nurse leaders. The plan is to combine a didactic program with role playing and make the scenarios as realistic as possible. A thorough debriefing of how difficult case situations are managed in simulated exercises will give the participants an opportunity to interact with each other, debrief in a group setting, and come up with several ways to handle a difficult situation. This will help charge nurses in the development of key leadership competencies needed in their roles. 

### 5.3. Relationship of the Findings to IOM Recommendations

The IOM Future of Nursing Report [1] has generated great interest among nurse leaders in the United States. One of four key messages in the report is that nurses should be full partners, with physicians and other health professionals, in redesigning health care in the United State. In order to achieve this vision, the report outlines a need to produce leaders throughout the system, from the bedside to the boardroom. It is suggested that being a full partner in care environments involves taking responsibility for identifying problems and implementing plans for improvement. Leadership will be fundamental to advancing the profession of nursing. The report recommends that leadership development must be available for nurses at all levels and individual nurses should take responsibility for their own personal and professional growth by developing leadership competencies. Berwick [14], in a response to the report, suggested that nursing is well positioned to be a change agent in the current health care delivery system but will require additional skill sets to lead and improve systems of care. Thibault [15] observed that understanding, mutual respect, and close working relationships between nurses and physicians will be critical to achieving the goals outlined in the IOM report. Fairman and Okoye [16] have noted that the IOM committee took a strong stand in the report on the need for better educated nurses to achieve reform, recommending that 80% of nurses earn baccalaureate degrees by 2010. 

There is an assumption in the IOM report that nurses will assume expanding leadership responsibilities given the right education and skill set. The feedback from the charge nurses in this study and other research conducted by one of the authors [17] indicates that many nurses have ambivalent feelings about leadership positions. O'Neil [18], in response to the IOM report, recommended that every nursing graduate should leave their educational setting with both the skill and expectation to provide leadership for the new healthcare delivery system. He cogently observed that without these expectations and image of themselves, no blue ribbon panel will make them become leaders.

The Tenet experience with charge nurse development and their lessons learned can provide guidance for leaders in other settings. The challenges of their charge nurses are similar to what has been reported in the research literature from other settings [5–9]. The recommendation in the recent IOM report that nurses especially those working at the front line of care develop strong leadership skills is of key importance in today's healthcare environment. With rising patient acuity, decreased lengths of stay, staffing shortages, pay for performance initiatives, and complex technologies, frontline nurses who assume charge nurse roles in acute care environments take on challenging responsibilities often without the benefit of any formal training. An investment needs to be in the ongoing education and competency development of charge nurses. 

## 6. Conclusion

Charge nurses in today's health care environment confront many challenges in their daily work. Excellent communication skills and the ability to effectively manage conflict are key qualities to achieving role success and maintaining a safe environment for patients. Developing staff and having an impact on patient outcomes are significant role satisfiers. This study indicates that many charge nurses are ambivalent about seeking higher level leadership positions. They are interested in developing their own leadership skills and highly value professional development opportunities offered by their nurse leaders.

This paper has provided guidance to Tenet Health System nurse leaders on what educational programming is needed for charge nurse development. Early outcomes from the ongoing educational development that Tenet has provided to their charge nurses are very promising. Part of the success has been driven by the commitment of senior nurse leaders. The recent IOM report has provided additional guidance regarding goals that should be established for charge nurse development. The journey to develop frontline nurse leaders is just beginning. 

## Figures and Tables

**Table 1:** Frontline leader demographics *N* = 354.

Age	Mean = 46.4 years of age SD = 5.7	
Gender	Female 322 (91%)	
	Male 32 (9%)	

Highest level of nursing education (degree)	Associate	156 (44%)
	Baccalaureate	134 (38%)
	Diploma	39 (11%)
	Masters	25 (7%)
	Doctorate	0

Years of nursing experience	Mean = 19.5 Years SD = 6.4	

Practice setting	26% Critical care	
	22% telemetry	
	16% medical-surgical unit	
	11% emergency room	
	7% obstetrics/gynecology	
	5% operating room	
	2% oncology	
	2% pediatrics	
	11% other	

**Table 2:** Leadership qualities needed by frontline nurse leaders.

Theme	Qualities	Frequency mentioned
Manages communication	Listening skills	293
	Keeping everyone updated	
	Sensitivity to communication styles	
	Confronts conflict directly	

Acts as the team coach	Clinical competence	243
	Seen as a go-to person	
	Expert educator	
	Cheer leader for the team	
	Team player	
	Collaborative	
	Knows how to delegate	

Seen as approachable	Nonjudgmental	183
	caring	
	Demonstrates empathy	
	Positive corrective feedback	
	Transparent	
	Available	

Works like an air traffic controller	Organizes the work of the team	168
	Ability to prioritize	
	Reduces unit chaos	
	Multitasks	
	Manages stress	

Viewed as a professional	Confident	123
	Assumes accountability for actions	
	Diplomacy with interdisciplinary team	
	Role model	
	Leadership respected by all	
	Professional advocate for nursing	

**Table 3:** Most challenging role responsibilities.

Challenging role responsibilities	Frequency checked
Managing conflict on the team	313
Keeping patients and families satisfied	225
Staying current with changes in policies and procedures	203
Delegating care to others	119
Ensuring good communication with team members	115
Meeting regulatory requirements	112
Maintaining a safe patient care environment	105
Making staffing decisions	95
Supervising the work of others	92
Coaching and giving feedback	92
Facilitating education and orientation	86
Communicating with physicians and consulting physicians	76
Making patient care assignments	58
Acting as preceptor for new staff	54
Arranging patient transfers	39
Other	26

**Table 4:** Most satisfying aspects of the frontline leadership role.

Theme	Satisfying aspects	Frequency mentioned
Developing staff	Coaching staff	90
	Watching new graduate grow	
	Teaching others	
	Serving as a mentor	

Keeping patients happy	Satisfied patients/families	87
	Good patient outcomes	

Leading the team	When team functions well	79
	When staff are happy	
	Positive feedback on leadership skills	

Making a difference	Solving problems	62
	Keeping things from falling through the cracks	
	Avoiding near misses	

Managing unit flow	Trouble shooting problems	40
	Avoiding chaos	
	Organizing care and staffing	
	Resolving conflict	

Becoming a leader	My personal growth	29
	Serving as a role model	
	Having autonomy	
	Being a good communicator	

Maintaining quality	Keeping patients safe	10
	Meeting all the measures	
	Caring about quality	

**Table 5:** Factors that would stop the participant from applying for the nurse manager role.

Theme	Factor subthemes	Frequency mentioned
Role compensation	Loss of salary	50
	Lack of job security	
	Loss of the 12-hour shift	
	Unpaid hours expected	
	Loss of overtime/shift differentials	

Role stress	Role expectations from staff, management,	50
	Patients, and families	
	Role responsibilities	
	Organizational politics	
	Regulatory/compliance accountability	
	Paperwork and budget	

Leaving the bedside	Loss of clinical skills	41
	Loss of direct patient care contact	
	Loss of satisfaction from patient care	

Role qualifications	Lack of academic education	39
	Lack of advanced leadership skills	
	Lack of self-confidence to lead at that level	

Role support	Anticipated lack of support from leaders	35
	Sandwiched between leadership & staff	
	No organizational mentors	

Outside support	Significant home and personal obligations	22
	Childrearing	
	Lack of support from spouse	
